# Coronary Plaque Boundary Enhancement in IVUS Image by Using a Modified Perona-Malik Diffusion Filter

**DOI:** 10.1155/2014/740627

**Published:** 2014-11-24

**Authors:** S. Anam, E. Uchino, N. Suetake

**Affiliations:** ^1^Graduate School of Science and Engineering, Yamaguchi University, Yamaguchi 753-8512, Japan; ^2^Mathematics Department, University of Brawijaya, Malang 65145, Indonesia; ^3^Fuzzy Logic Systems Institute, Iizuka 820-0067, Japan

## Abstract

We propose a modified Perona-Malik diffusion (PMD) filter to enhance a coronary plaque boundary by considering the conditions peculiar to an intravascular ultrasound (IVUS) image. The IVUS image is commonly used for a diagnosis of acute coronary syndrome (ACS). The IVUS image is however very grainy due to heavy speckle noise. When the normal PMD filter is applied for speckle noise reduction in the IVUS image, the coronary plaque boundary becomes vague. For this problem, we propose a modified PMD filter which is designed in special reference to the coronary plaque boundary detection. It can then not only reduce the speckle noise but also enhance clearly the coronary plaque boundary. After applying the modified PMD filter to the IVUS image, the coronary plaque boundaries are successfully detected further by applying the Takagi-Sugeno fuzzy model. The accuracy of the proposed method has been confirmed numerically by the experiments.

## 1. Introduction

**Figure 1:**
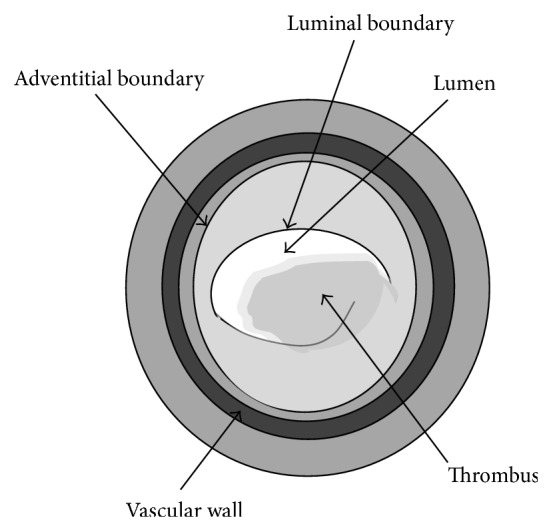
Illustration of ruptured plaque.

Acute coronary syndrome (ACS), which is commonly known as atherosclerotic heart disease, is the most common type of heart disease and the cause of heart attacks. Recently, ACS became one of the leading hospitalizations in the world. ACS is a term used to describe any condition that results in a sudden reduction in blood flow to the heart. ACS happens when the plaques are built up inside the coronary arteries. If the plaque ruptures as shown in Figure 1, it can lead to a blood clot that blocks the blood supply to the heart and triggers a heart attack. It may also block blood supply to the brain, which could trigger a stroke.

Intravascular ultrasound (IVUS) imaging [1] is a unique imaging clinic tool that provides a real time cross-sectional inside view of a coronary artery in living individual and thus allows a complete study of its morphology, such as arterial wall, lumen, and plaque. The IVUS method helps in the diagnosis and treatment of ACS, as far as a tissue characterization and plaque volume calculation are available. As the first step in a diagnosis of ACS, the inner and outer coronary plaque boundaries in the IVUS image have to be detected for evaluating the quantitative assessment of the coronary plaque compositions.

Currently, these coronary plaque boundaries are manually drawn and the area of that plaque is also evaluated manually by a medical doctor. After that, the volume of the plaque is estimated by integrating the calculated areas. A manual processing of the IVUS images by a medical doctor is a time consuming task and a difficult task and might suffer from intra- and interobserver variability. This is not only because a large number of the IVUS images must be processed, but also because the IVUS images tend to a heavy speckle noise. This fact motivates the development of automatic image processing technique addressing detection of the coronary plaque boundary with high accuracy.

Several algorithms for the coronary plaque boundary have been proposed [2–6]. A probabilistic segmentation for identification of luminal boundary (LB) has been proposed by Ruiz et al. [2]. Gil et al. [3] presented a statistical strategy for anisotropic adventitial modelling. Those methods however do not automatically work because the method [2] needs an initial area created by a user, and the method [3] needs a set of training data manually segmented by an expert. Unal et al. [4] has proposed a shape driven segmentation method. This method also needs a set of training data which is manually segmented by an expert.

In our previous works [5, 6], the fuzzy inference based method was proposed and applied for this problem. Fuzzy inference model employed is Takagi-Sugeno (T-S) fuzzy model [7]. In [5], membership functions (MSFs) in the antecedent parts of the fuzzy rules were allocated adaptively. The fuzzy inference was used because it has several advantages over the conventional methods in the boundary calculation of image, for example, Sobel's method, Prewitt's method, and Robert's method [8]. The fuzzy inference can handle problems with imprecise, noisy, inconsistent, and incomplete data set [9]. IVUS images often have noise and the plaque boundaries are usually missing in several areas. We have employed the T-S fuzzy inference to restore the missing boundaries by inference.

Because the IVUS image has heavy speckle noise, a speckle noise reduction and an edge enhancement of coronary plaque boundary are very important tasks in the case of preprocessing of IVUS image. As the representative conventional noise reduction methods, the median filters [10], morphology analysis [11], and bilateral filters [12] are well known, but at the same time the coronary plaque boundary also becomes dull unexpectedly by applying those methods.

Above all those methods, Perona-Malik diffusion (PMD) filter [13] is known as an effective edge-preserved smoothing method and is broadly used in [3, 5, 6, 13–18]. In [5, 6], the normal PMD filter is used to reduce the speckle noise. However, when the normal PMD filter is applied to IVUS image, the coronary plaque boundary cannot be preserved on several areas. The diffusion direction and its strength are very important factors to enhance the image edges and to reduce the speckle noise. This is because the diffusion direction and its strength in the normal PMD filter used in [5, 6] have not been set properly and the plaque boundary direction in IVUS image was not considered.

In this paper, we propose a modified PMD filter to reduce the speckle noise and enhance a coronary plaque boundary by considering the plaque direction in IVUS image. After applying the modified PMD filter to IVUS image, the coronary plaque boundaries are detected by the Takagi Sugeno (T-S) fuzzy model [7]. The accuracy of the proposed method has been verified through the experiments using the real IVUS images.

## 2. Intravascular Ultrasound Image

The intravascular ultrasound (IVUS) method is one of the applications of ultrasound technology which has many applications in medical diagnosis. The IVUS method is a catheter based medical imaging technique that produces cross-sectional images of blood vessel and is particularly useful for the diagnosis of atherosclerosis [19].

IVUS method is further used in the coronary arteries to observe within the blood vessel all the way through to the surrounding blood column, visualizing the coronary plaque, determining the amount of plaque built up at any particular point in the coronary artery in living individual. The progressive accumulation of plaque within the artery wall over decades is the setup for vulnerable plaque which, in turn, leads to heart attack and stenosis of the artery.

**Figure 2:**
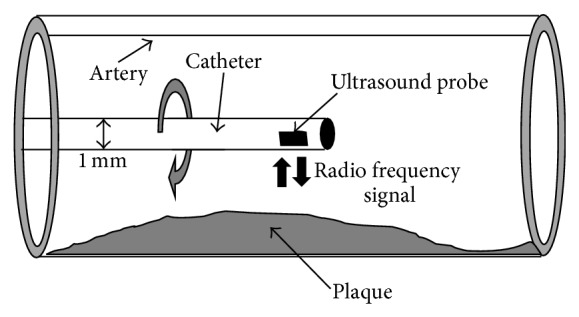
An ultrasound probe attached to the distal end of a catheter.

The IVUS method uses a specially designed thin catheter with the ultimately miniaturized ultrasound probe attached to its distal end (see Figure 2). The probe rotates in the arterial lumen in order to receive an ultrasound radio frequency (RF) signal reflected from the plaque and the vascular wall.

The IVUS images are acquired by means of high-frequency, single-use probes based on various mechanical and electronic phased array systems. The IVUS image is generated by using the amplitude information from the received ultrasound RF signals.

In the first step, the sampled RF signal is first transformed into an 8-bit luminal intensity signal in all radial directions by taking the absolute value of the signal. As the second step, because the RF signal propagating in the tissue is affected by attenuation due to depth, it is necessary to compensate it by time gain compensation function. As the third step, in order to reduce the noise effect and spurious harmonic components outside the band of interest, the RF signal can be filtered by a band-pass filter. After taking the envelope of this signal, data is normalized. As the last step, in order to expand the effective dynamic range of our digital images in terms of saturation, we can optionally apply the gamma correction using the gamma value, the gradient of the linear region on the gamma value curve [20].

**Figure 3:**
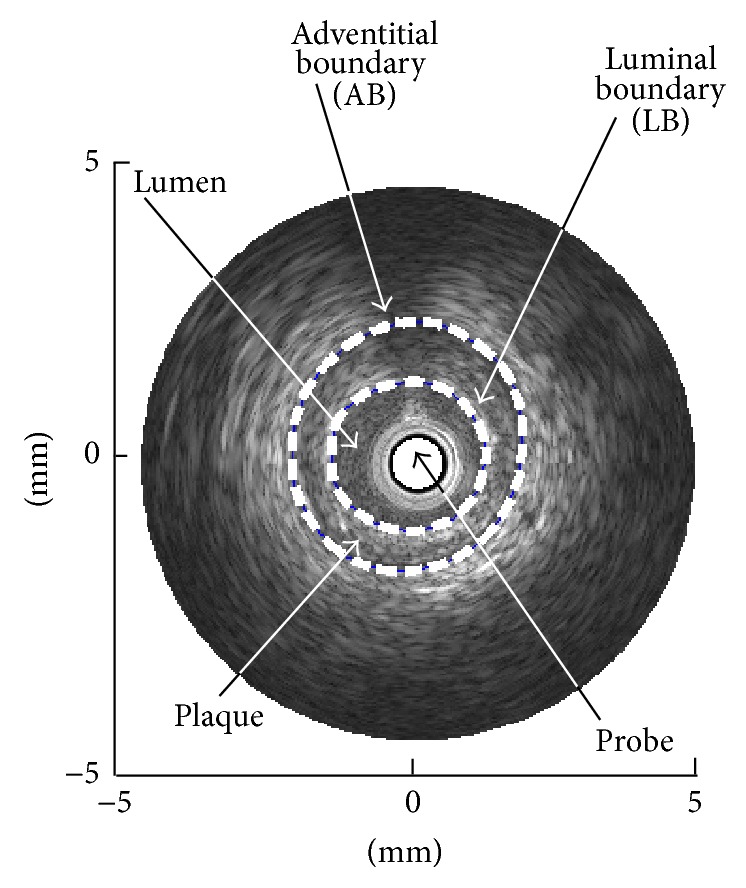
B-mode image in the Cartesian coordinates.

As the final result, we get a tomographic cross-sectional image of a coronary artery as shown in Figure 3. This image is called a “B-mode image.” A B-mode image displays a real time ultrasound cross-sectional image of a thin section of a blood vessel where currently a catheter probe is rotating.

In quantitative assessment of coronary plaque, the following two boundaries are calculated in the IVUS B-mode image. One is a luminal boundary (LB) between the lumen and the plaque, and the other is an adventitial boundary (AB) between the plaque and the vascular wall.

## 3. Image Separability

Many edge extraction methods have been proposed, most of which are mainly based on the gradient of image intensity. These gradient-based methods use the smoothing filter such as a Gaussian filter for suppression of noise. Since they blur edges, the precision for edge localization degrades.

An edge detection method by using a statistical discriminant measure called image separability has been proposed by Fukui [21]. This method has advantages over the gradient based methods, because the image separability has the following features:

- insensitivity to noisy and blurred edges,
- ability to differentiate the edges between texture regions.

For this reason, to distinguish the difference between the plaque boundary and the speckle noise in an IVUS image, the proposed method employs the image separability.

**Figure 4:**
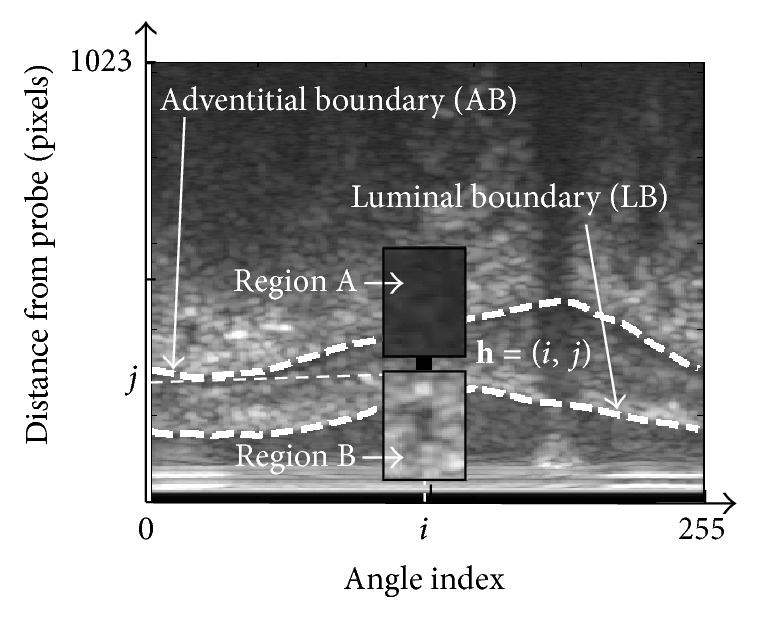
Detection of plaque boundary by using image separability.

Figure 4 shows a local region which consists of two small regions *A* and *B*. The separability *η*_**h**_ for pixel **h** can be calculated by linear discriminant analysis with information from regions *A* and *B* as follows:

(1) $$\begin{array}{c} \eta_{h}=\frac{n_{A}{\left({\overset{-}{I}}_{A}-\overset{-}{I}\right)}^{2}+n_{B}{\left({\overset{-}{I}}_{B}-\overset{-}{I}\right)}^{2}}{\sum_{k=1}^{S}{\left(I_{k}-\overset{-}{I}\right)}^{2}}, \end{array}$$

where *n*_*A*_ and *n*_*B*_ represent the numbers of the pixels in the regions of *A* and *B*, respectively. ${\overset{-}{I}}_{A}$ and ${\overset{-}{I}}_{B}$ represent the averages of intensities in the regions of *A* and *B*. $\overset{-}{I}$ stands for the average of the intensities in the combined regions of *A* and *B*. *S* and *I*_*k*_ represent the number of the pixels and the intensity of the *k*th pixel in the combined regions of *A* and *B*.

The weighted separability for pixel **h**, which is a modification of the original separability [6], is defined by

(2) $$\begin{array}{c} \eta_{h}^{w}=\eta_{h}{\left(\frac{I_{\max}-{\overset{-}{I}}_{A}}{I_{\max}}\times \frac{{\overset{-}{I}}_{B}}{I_{\max}}\right)}^{2}, \end{array}$$

where *I*_max⁡_ is the maximum intensity in the whole of IVUS image and *η*_**h**_^*w*^ satisfies 0 ≤ *η*_**h**_^*w*^ ≤ 1. The image separability takes a larger value when two regions are separated from each other.

The weighted image separability detects the candidates of the inner and outer boundaries of plaque by considering the following two conditions peculiar to IVUS images:

- intensity in the outside area of a luminal boundary (LB) tends to be stronger than that in the inside area of LB,
- intensity in the outside area of an adventitial boundary (AB) tends to be stronger than that in the inside area of AB [22].

## 4. Proposed Method

We present in this paper a modified PMD filter to reduce speckle noise and enhance a coronary plaque boundary in IVUS image. This chapter is divided into two sections which are (i) modified Perona-Malik diffusion (PMD) filter and (ii) boundary calculation procedure by fuzzy inference.

### 4.1. Modified Perona-Malik Diffusion (PMD) Filter

Perona and Malik have proposed an anisotropic diffusion filter, which is known as Perona-Malik diffusion (PMD) filter, to filter noise and preserve the edges of an image. The basic idea of the PMD process is to get an increasingly smoothed image *u*(*x*, *y*, *t*) from an original image *u*_0_(*x*, *y*), indexed by diffusion parameter *t*. This process can be interpreted as an image convolution by a Gaussian kernel *G*(*x*, *y*, *t*) with an increasing width as follows:

(3) $$\begin{array}{c} I\left(x,y,t\right)=I_{0}\left(x,y\right)*G\left(x,y,t\right). \end{array}$$

The PMD filter equation is defined by

(4) $$\begin{array}{c} I_{t}=\frac{\partial I}{\partial t}=\mathrm{div}\left(c\left(x,y,t\right)\nabla I\right)=c\left(x,y,t\right)\Delta I+\nabla c\left(x,y,t\right)\nabla I, \end{array}$$

where

(5) $$\begin{array}{c} c\left(x,y,t\right)=g\left(\left\|\nabla I\left(x,y,t\right)\right\|\right) \end{array}$$

is a diffusion coefficient. ∇*I* denotes a gradient of an image.

*g*(·) refers to an edge stopping function, which is a decreasing function of the gradient of image, which is defined by

(6) $$\begin{array}{c} g\left(\nabla I\right)=\frac{1}{1+{\left(\nabla I/K\right)}^{2}}, \end{array}$$

respectively, where *K* is a parameter which controls the strength of diffusion. *g*(·) takes large values at the regions where the intensity gradients are low. On the contrary, it takes low values at the regions where the intensity gradients are high.

The initial condition is given by

(7) $$\begin{array}{c} I\left(x,y,0\right)=I_{0}\left(x,y\right). \end{array}$$

**Figure 5:**
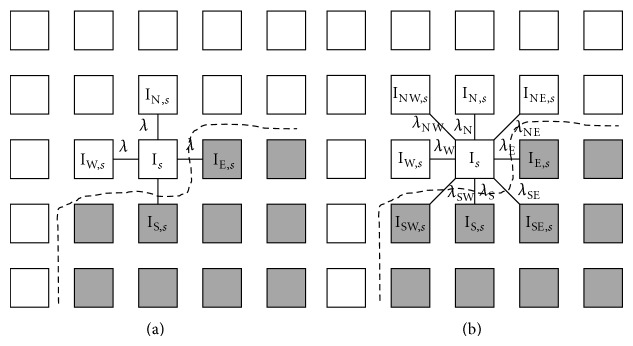
Structure of diffusion directions. (a) Normal PMD filter. (b) Modified PMD filter.

The discrete version of PMD process is defined as follows:

(8) $$\begin{array}{c} I_{s}^{\left(n+1\right)}=I_{s}^{\left(n\right)}+\frac{\lambda }{\left|\phi_{s}\right|}\sum_{}g\left(\nabla I_{s,p}^{\left(n\right)}\right)I_{s,p}^{\left(n\right)}, \end{array}$$

where *s* = (*x*, *y*) and *p* are the coordinates of the pixel of concern and its neighboring pixels, respectively. *I*_*s*_^(*n*)^ is an intensity at *s* with an iteration count *n*. *ϕ*_*s*_ represents the four neighboring pixels in north, west, south, and east diffusion directions. |*ϕ*_*s*_| is the number of pixels in the neighborhood area. *λ* is a parameter. The structure of diffusion direction for the normal PMD filters is shown in Figure 5(a).

However, when the normal PMD filter is applied to IVUS image, the coronary plaque boundary cannot be preserved on several areas. By analyzing many experiments, we can conclude that the diffusion direction and its strength are very important factors in the PMD filter to enhance the edge of image and to reduce the noise.

If the strength of diffusion is too large, the edge of the image tends to be lost. On the contrary, if the strength of diffusion is too small, the noise of image cannot be reduced. When the diffusion direction and its strength are set properly, the PMD filter can enhance the plaque boundary and reduce noise. Therefore, the direction and strength of diffusion must be set properly for a good filtering performance.

We propose here the modified direction and strength of diffusion of the PMD filter by considering the plaque boundary direction in the IVUS image. From Figure 4 it can be observed that the boundaries of plaque are in horizontal direction. It means that in order to preserve the plaque boundaries, the diffusion strength in horizontal direction should be smaller than that in other directions. In order to consider flexibly the direction of plaque boundary, we propose a new structure for diffusion directions as shown in Figure 5(b).

The modified PMD filter moves in eight directions with different strength in each direction. By modifying the diffusion process of the normal PMD filter of (8) based on the diffusion direction in Figure 5(b), the proposed iteration formula for diffusion process of the modified PMD filter is given as follows:

(9) $$\begin{array}{c} I_{s}^{\left(n+1\right)}=I_{s}^{\left(n\right)}+\frac{1}{\left|\phi_{s}\right|}\sum \lambda_{k}g\left(\nabla I_{k,s}^{\left(n\right)}\right)I_{k,s}^{\left(n\right)}, \end{array}$$

where *k* = {NW, N, NE, E, SE, S, SW, W}. NW, N through W represent the direction of northwest, north, northeast, east, southeast, south, southwest, and west, respectively [23].

### 4.2. The Boundary Calculation Procedure by Fuzzy Inference

The boundary calculation procedure is briefly summarized as follows.

**Figure 6:**
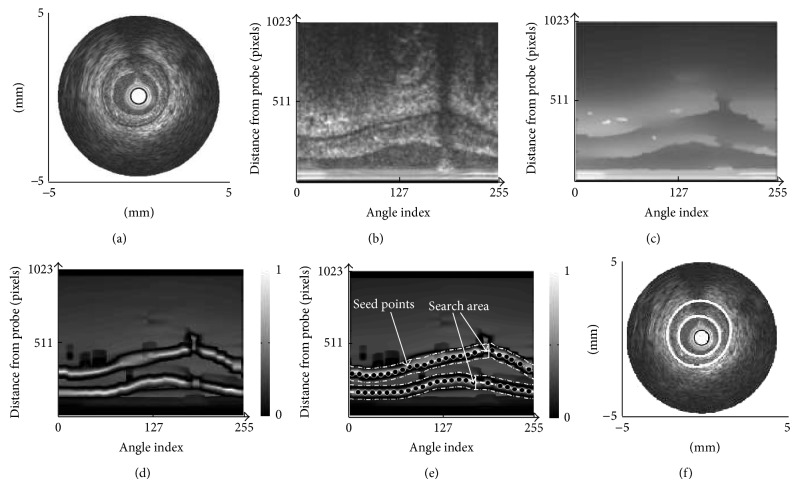
Boundary calculation procedure. (a) IVUS image in Cartesian coordinate. (b) Transposed image of (a) into polar coordinate. (c) Filtered image of (b) by modified PMD filter. (d) Image separability of (c) by weighted image separability. (e) Search area by placed automatic seed points. (f) Plaque boundary calculation results by fuzzy inference.

- A B-mode image is transform from Cartesian coordinate shown in Figure 6(a) into polar coordinate shown in Figure 6(b).
- The B-mode image in polar coordinate is filtered by modified PMD filter of (9) whose filtering result is shown in Figure 6(c).
- An image separability of Figure 6(c), whose result is shown in Figure 6(d), is calculated.
- Seed points are roughly placed automatically on the B-mode image to obtain search areas as shown in Figure 6(e) described in [2].
- The plaque boundary is inferred by using the Takagi-Sugeno (T-S) fuzzy model described in [5].
- The plaque boundary results inferred by T-S fuzzy model are shown in Figure 6(f).

## 5. Experimental Results

**Figure 7:**
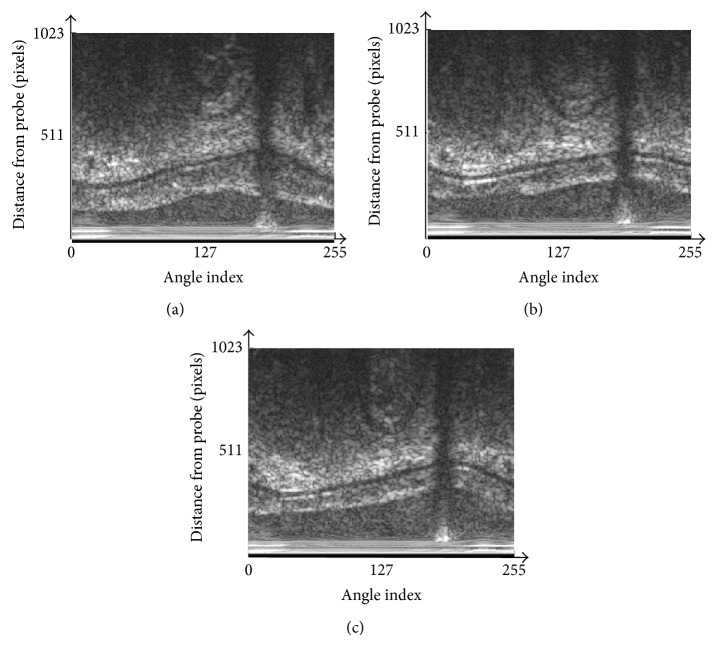
IVUS image in polar coordinate to be processed. (a) Image 1. (b) Image 2. (c) Image 3.

In the experiments, we use three different IVUS images which are shown in Figure 7, and the proposed method is compared with the method using the normal PMD filter in [6].

By analyzing for many IVUS images, the parameters of the diffusion filter of (9) were set as *λ*_N_ = *λ*_S_ = 1 and *λ*_W_ = *λ*_E_ = *λ*_NW_ = *λ*_SW_ = *λ*_SE_ = *λ*_NE_ = 1.2.

**Figure 8:**
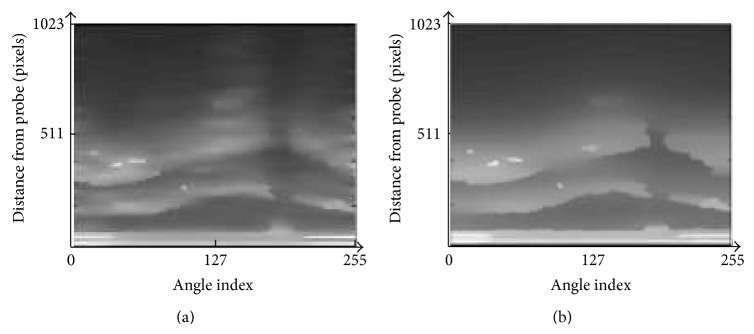
Diffusion filter results for image 1. (a) The normal PMD filter [13]. (b) The modified PMD filter.

**Figure 9:**
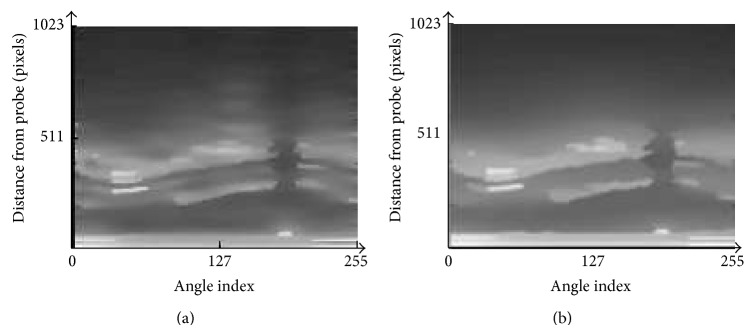
Diffusion filter results for image 2. (a) The normal PMD filter [13]. (b) The modified PMD filter.

**Figure 10:**
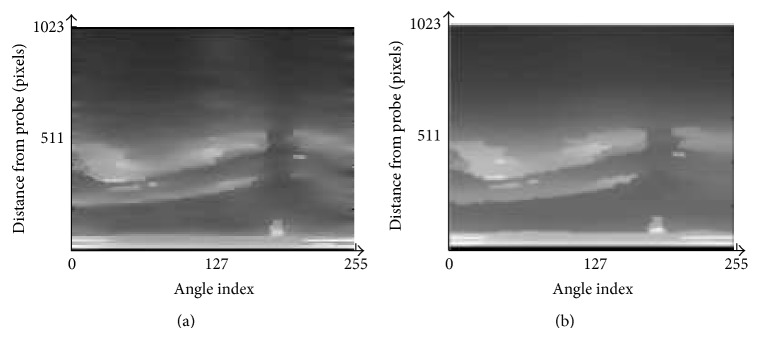
Diffusion filter results for image 3. (a) The normal PMD filter [13]. (b) The modified PMD filter.

Figures 8(a), 9(a), and 10(a) show the filtering results by the normal PMD filter [13]. Figures 8(b), 9(b), and 10(b) show the filtering results by the modified PMD filter. The plaque boundaries by the modified PMD filter are more clearly enhanced than by the normal PMD filter [13]. It is indicated that the modified PMD filter is better than the normal PMD filter in the plaque boundary enhancement.

**Figure 11:**
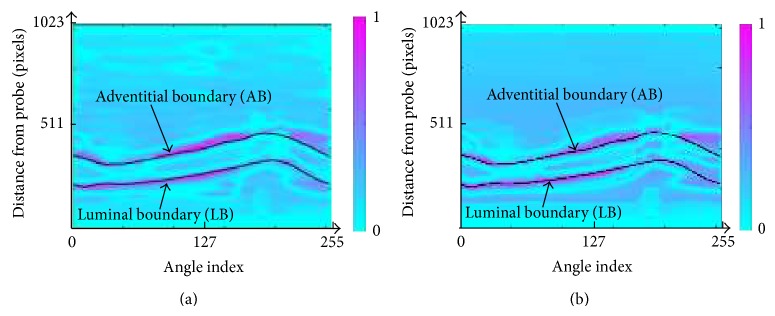
Weighted image separability for image 3. (a) The method with the normal PMD filter [13]. (b) The method with the modified PMD filter.

Figures 11(a) and 11(b) show the weighted image separability of image 3 after applying the normal PMD filter and after applying the modified PMD filter, respectively. The red areas in Figures 11(a) and 11(b) show the possible areas where the desired (true) boundaries exist. The desired (true) boundaries are detected by the experts, and they are indicated by the black solid lines in both figures. We can see that the red areas of Figure 11(b), obtained by using the present modified PMD filter, catch the desired (true) boundaries in the center of red areas better than using the normal PMD filter.

The root mean square errors (RMSEs) between the desired and the detected plaque boundaries are shown in Table 1. The RMSEs of the proposed method are better than those of the method in [6] using the normal PMD filter for the most parts of LB and all parts of AB.

**Figure 12:**
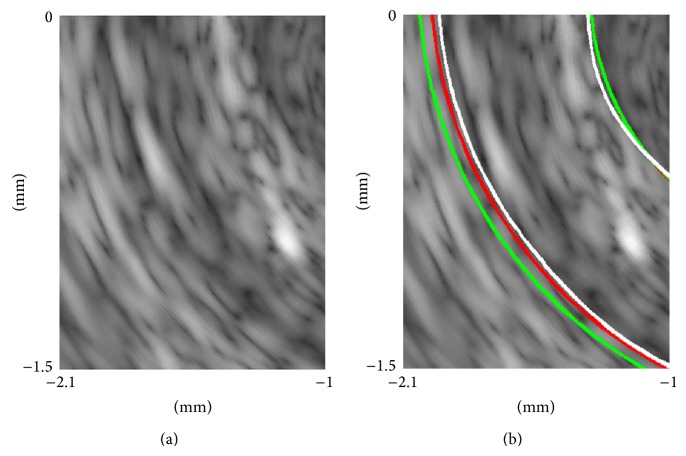
Comparison of plaque boundary detection. (a) IVUS image 1 to be processed. (b) Boundary detection results. The red lines and the green lines indicate the boundaries detected by the proposed method and by the method with the normal PMD filter [6], respectively. The white lines are the desired boundaries.

The comparisons of plaque boundary detection for image 1 by the method in [6] with the normal PMD filter and by the proposed method are shown in Figure 12. The detected boundary by the proposed method (red line) is closer to the desired boundary (white line) than that by the method in [6] (green line). The accuracy of the proposed method is increased.

## 6. Conclusion

We have proposed a modified PMD filter to reduce speckle noise and to enhance the coronary plaque boundary in the IVUS image. After applying the modified PMD filter, the plaque boundaries were detected successfully by T-S fuzzy inference. The plaque detection accuracy was good in spite of the very noisy IVUS image. The results show that the proposed method is better than that of the method with the normal PMD filter.

## Figures and Tables

**Table 1:** RMSEs of boundary detection results (*μ*m).

Method	Image 1		Image 2		Image 3	
	LB	AB	LB	AB	LB	AB
Method with normal PMD [6]	13.7	28.4	**20.0**	30.2	28.1	35.4
Proposed method	**9.5**	**15.0**	20.3	**14.0**	**14.0**	**34.6**
